# Mixed-methods evaluation of Daily Moves, a community-based physical activity program for older adults

**DOI:** 10.1186/s12877-022-03567-6

**Published:** 2022-11-12

**Authors:** Maddison L. Mellow, Melissa J. Hull, Ashleigh E. Smith, Thomas P. Wycherley, Danielle Girard, Alyson J. Crozier

**Affiliations:** 1 Alliance for Research in Exercise Nutrition and Activity (ARENA), Allied Health and Human Performance, University of South Australia, Adelaide, Australia; 2 Allied Health and Human Performance Unit, University of South Australia, Adelaide, Australia; 3 UniSA Online, University of South Australia, Adelaide, Australia

**Keywords:** Healthy aging, Physical activity, Exercise, Wellbeing

## Abstract

**Background:**

Although the health benefits of physical activity are well documented, most older adults are not sufficiently active. There is a need to explore approaches to physical activity promotion amongst older adults that meet the personal preferences and needs of participants, and that can be implemented on a large scale in community-based settings. The current study evaluates *Daily Moves,* a community-based physical activity program for older adults living in Adelaide, Australia.

**Methods:**

The *Daily Moves* program, which ran almost entirely during the COVID-19 pandemic, provided participants with personalized plans and information about suitable physical activity promoting activities available in their local area. This study used an explanatory sequential mixed-methods approach to evaluate associations between participation in the Daily Moves program and physical activity engagement, physical function and psychosocial wellbeing, and to explore the experiences of Daily Moves participants through qualitative interviews, with a particular focus on the impact of the COVID-19 pandemic on program participation and enjoyment.

**Results:**

The research evaluation included 69 older adults (mean age at baseline = 73.9 ± 5.6 years; 19 male). Following *Daily Moves*, participants reported an increase in self-report physical activity levels (mean increase = 1.8 days, *p* < 0.001), improvements on several measures of physical function (left grip strength (mean increase = 1.8 kg, *p* < 0.001); right grip strength (mean increase = 1.3 kg, *p* = 0.03); Timed Up and Go (mean decrease = 1.3 s, *p* < 0.001)), and no significant changes in measures of psychosocial wellbeing. Qualitative interviews revealed that participants valued the supportive and flexible nature of *Daily Moves,* and that they felt connected with staff and other participants despite the onset of the pandemic.

**Conclusions:**

This evaluation demonstrates that physical activity programs embedded within the community can provide flexible and tailored recommendations to participants, and that this approach can promote positive change in important indicators of health in older adults.

**Supplementary Information:**

The online version contains supplementary material available at 10.1186/s12877-022-03567-6.

## Background

Staying physically active in older adulthood is beneficial for health. These benefits extend across many aspects of physical health, including reduced risk of cardiovascular disease, osteoporosis, falls, and other chronic diseases [1]. Higher levels of physical activity (such as walking, cycling or strength training) have also been linked to better mental wellbeing, quality of life, and lower rates of depression [2]. Although the benefits of physical activity are well-accepted, most older Australians are not sufficiently active against Australian guidelines [3]. Based on recent national survey data, only 26.1% of Australian older adults (65 years and above) engaged in 30 min of moderate physical activity on five or more days in the last week [3], and less than 15% of adults aged > 60 years met strength training guidelines (two sessions per week) [4]. In South Australia, results were similar with 34.1% of those aged 65 and over reporting daily physical activity engagement [5]. Taken together, there is a need for effective interventions which increase physical activity levels among older adults.

The implementation of community-based physical activity programs through government and health agencies in Australia have shown promise in increasing activity levels and improving physical and psychosocial wellbeing in community-dwelling older adults. Successful examples include the Strength for Life program [6], and the National Heart Foundation of Australia walking groups [7]. Such programs are commonly centered around synchronously increasing social and physical activity engagement, based on evidence that social engagement is positively associated with physical and psychosocial health [8]. However, many physical activity interventions have faced an “implementation-scale up gap” in which programs that were deemed successful on a small scale are not effectively expanded and implemented under real-world conditions [9]. Thus, there is a need to explore approaches to physical activity promotion amongst older adults that can be implemented on a large scale and in community-based settings effectively.

There are multiple factors that may influence adherence to prescribed physical activity, including the demographic of participants, prior experience and knowledge of physical activity, personal factors such as motivation and willingness to change, enjoyment, and perceived benefits and barriers to being physically active [10, 11]. The affordability and availability of activities or programs that promote physical activity engagement are also important determinants of physical activity engagement in older adults [12]. Physical activity interventions that overcome these barriers may influence the success of such interventions in community-based settings [11, 13]. These interventions need to meet the personal preferences and needs of participants and ensure that increasing physical activity can be achieved in the participant’s everyday setting to promote long-term change [13].

*Daily Moves* was a community-based program that aimed to increase physical activity levels among community-dwelling older adults living in six council areas in Adelaide, South Australia. The program was designed and implemented by the City of Unley council and funded by an Australian Sports Commission Better Ageing grant from 2019 to 2021, following an extension of the original 2-year program due to COVID-19 delays. This study used an explanatory sequential mixed-methods approach to: 1) quantitatively evaluate associations between participation in the Daily Moves program and physical activity engagement, physical function and psychosocial wellbeing, and 2) explore the lived experiences of Daily Moves participants with a particular focus on the impact of the COVID-19 pandemic on program participation and enjoyment, and 3) identify participant preferences and needs that were supported by the program which could inform development of similar future community based programs.

## Methods

### Research design

The study used an explanatory sequential mixed-methods approach, with data collected between September 2019 and June 2021 from Daily Moves program participants.

### Daily Moves program

Daily Moves was available to all people aged 65 years and older, who resided in one of six metropolitan councils in eastern Adelaide, South Australia (City of Burnside, City of Unley, City of Walkerville, City of Prospect, City of Norwood Payneham and St Peters, City of Campbelltown). The focus of Daily Moves was to promote an increase in physical activity levels of older adults by providing personalized plans and information about suitable physical activity promoting activities available in their local area (i.e., gym classes, local community centers, or readily available physical activity programs).

Participants were recruited to Daily Moves via widespread community advertisement (i.e., flyers placed in newsletters, social media profiles, libraries, and community halls). Interested participants were directed to contact Daily Moves staff at the City of Unley to register. Information was also offered to older adults registered on council databases. Following their registration, participants were provided with an information pack containing information about the Daily Moves program, a letter to provide to their General Practitioner (GP), and instructions on how to book their initial assessment session. All participants required medical clearance from their GP prior to attending their baseline assessment.

**Fig. 1:**
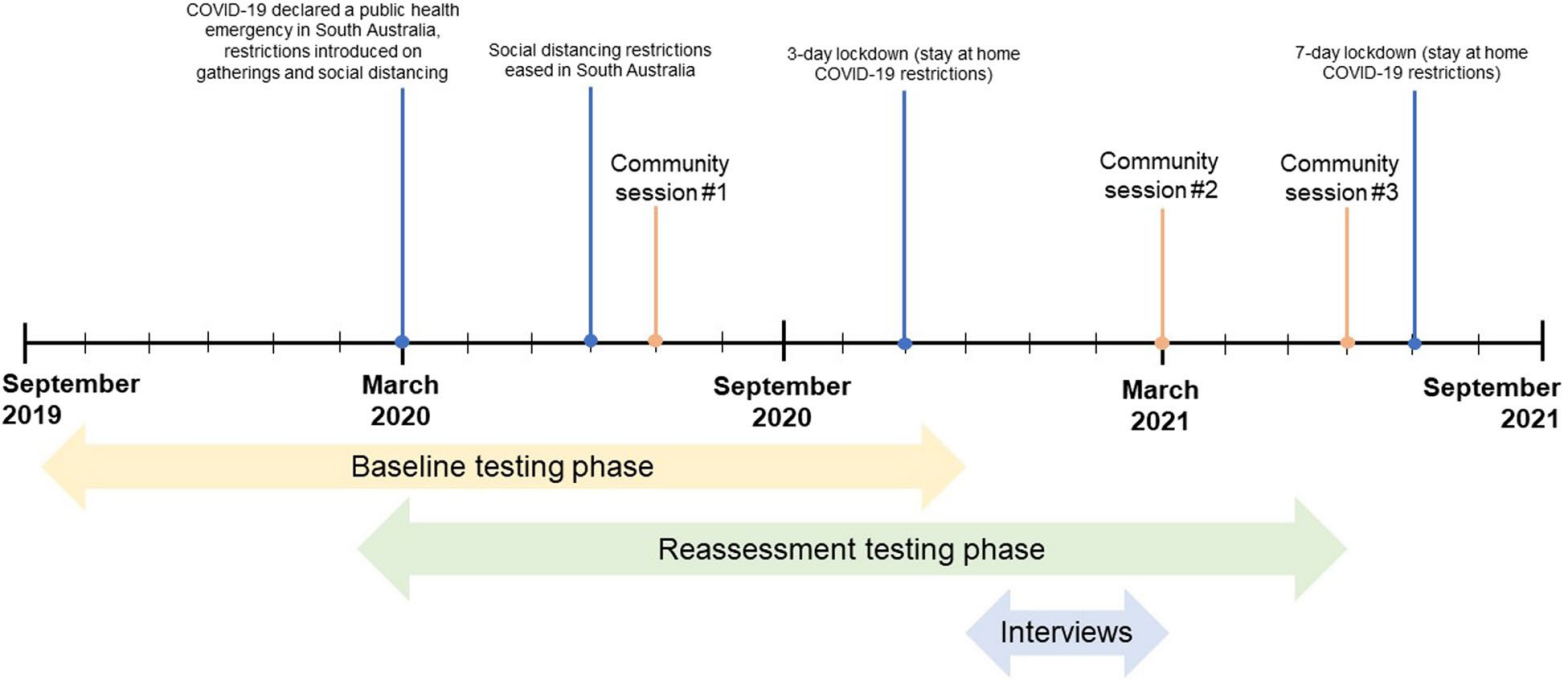
Conceptual model of Daily Moves program

To assess potential changes in physical function and psychosocial wellbeing measures in Daily Moves participants, two optional assessment sessions (baseline and reassessment) were conducted between September 2019 and June 2021 (Fig. 1). Participants were able to attend either a community-based assessment session held in community halls and recreation facilities, or a private assessment in their own home. All assessment sessions were supervised by an Exercise and Sports Science Australia Accredited Exercise Scientist. Undergraduate students on work-integrated learning placements across Exercise Science, Social Work and Psychology supported the delivery of the physical and psychosocial assessments to participants. After their baseline assessment, participants engaged in a motivational interview to discuss goals and interests around physical activities they would like to engage in. All staff and students conducting assessments were trained and inducted on the standard application of the assessments before working directly with participants.

Figure 1 outlines the timeline of the Daily Moves program and COVID-19 interruptions during the program in South Australia. Baseline and reassessment testing phases include quantitative physical and psychosocial assessments. Community sessions were held in July 2020, March 2021 and June 2021.

Based on their baseline assessment session and motivational interviews, individualized Daily Moves plans were developed, considering participant’s preferences, capabilities, and location. Participants then used these plans to support their daily physical activity routine to increase both incidental and planned bouts of physical activity and movement throughout their day. Some home exercise equipment (e.g., dumbbells and resistance bands) were available to participants for loan. Throughout the program participants also had optional access to volunteers and free 1:1 support from qualified practitioners (e.g., exercise physiologists, personal trainers, support workers) on a needs basis. Optional community wide information sessions were held on a range of topics related to healthy ageing throughout the program (e.g., sleep, supermarket tours, using outdoor park equipment).

Throughout the program, two staff members from the City of Unley were engaged as project officers and were responsible for the day-to-day implementation, scheduling and administration of the program. Participants were directed to contact a central phone number or email if they required any additional support, equipment, or re-assessment (outside the standard baseline and reassessment visits) throughout the program.

### COVID-19 impacts on program delivery

Due to the outbreak of COVID-19, some aspects of Daily Moves were delayed or unable to run in their entirety as initially planned. Social distancing requirements meant assessment sessions were postponed for several months. Therefore, baseline assessments often did not occur as participants entered the program, but instead 2–3 months after (i.e., when restrictions had eased). Many group-based activities including community activity classes and demonstrations were indefinitely postponed during this time.

To suit the need to deliver activities via virtual methods, additional video-based resources including short video demonstrations and classes were recorded and uploaded to the Daily Moves YouTube channel (https://www.youtube.com/channel/UChkWzVYthGdA9puCAtCpbwA). Daily Moves staff were also engaged in a supportive role following the announcement of the COVID-19 pandemic, and phoned participants regularly to support their wellbeing during this time. These supports included connection to wider council services, a friendly conversation or direction to additional movement resources depending on participant preferences and needs.

During initial planning of evaluation, our intention was to conduct assessments at baseline, 6-months and 12-months. As a result of COVID-19, a singular follow-up period at approximately 9-months was established to pragmatically capture changes from baseline to reassessment. Qualitative interviews were added to the evaluation in response to the COVID-19 pandemic to understand the impacts on participants, their activity choices and wider motivations to continue to be active during this time.

### Ethics approval and consent for evaluation

Ethics approval was provided by the University of South Australia’s Human Research Ethics Committee (Approval number 00000202748), allowing researchers to evaluate the effect of the program on health and wellbeing outcomes. Participants were provided an information sheet about the evaluation when they registered for Daily Moves. At their baseline assessment session, participants were offered an opportunity to provide informed written consent to the Daily Moves project team for their data to be included in this evaluation. Recorded informed consent was then obtained prior to the beginning of the interview. If participants did not consent to having their data included in the research evaluation, their participation in Daily Moves was not affected, and their data were still provided to the City of Unley council for internal evaluation of the effectiveness of the program. All procedures were conducted in accordance with the Declaration of Helsinki.

### Evaluation design and procedures

The evaluation of Daily Moves was conducted in two phases: a quantitative assessment of change in physical and psychosocial measures, followed by qualitative interviews exploring the experiences of participants in the program following COVID-19.

### Measures of physical wellbeing

To evaluate the outcomes of the Daily Moves program, we measured physical activity engagement, physical function and performance, and psychosocial wellbeing at baseline (beginning of program) and reassessment (~ 9 months into program). Engagement in physical activity at both timepoints was assessed using a self-report single item measure. At baseline and reassessment, participants were asked “In the past week, how many days have you done a total of 30 min or more of physical activity, which was enough to raise your breathing rate? (This may include sport, exercise and brisk walking or cycling for recreation or to get to and from places but should not include housework or physical activity that is part of your job)”. Potential scores ranged from 0 to 7 days [14]. This measure has previously demonstrated moderate correlations with accelerometer-based measured of moderate-vigorous physical activity engagement in cohorts of similar age [15].

Four valid and reliable tests of physical function and performance were also administered at baseline and reassessment timepoints: Grip Strength test [16]; Unipedal Stance Test [17]; 6-Minute Walk Test (6MWT) [18]; and Timed Up and Go test [19].

The Grip Strength test [16] required participants to perform a maximal single muscular contraction on a hydraulic hand dynamometer (Jamar, model 5030J1) for three trials each with the left and right hand. The best grip strength score (in kilograms) for each hand was recorded to the nearest 0.1 kg. The Grip Strength test is used as an indicator of overall muscular strength capacity and has demonstrated high test–retest reliability in community-dwelling older adults [20].

The Unipedal Stance test [17] is used to assess postural control and balance and can be an indicator of falls risk, and has demonstrated good test–retest reliability in older adults (ICC = 0.78) [21]. Three separate trials were performed with eyes open, and three with eyes closed. The aim of the task was to remain in position (balanced on one leg, arms crossed, with eyes open/closed) for as long as possible (up to 45 s). Each attempt was recorded to the nearest 0.1s, and the best attempt was determined for eyes open, and eyes closed.

The 6MWT [18] is designed to measure physical capacity and functional performance. A modified version of the 6MWT was performed using a shorter course than the typical 30-m course (10 m, due to space constraints). Participants were timed for 6 min as they walked between two marked lines, and standardised encouragement was provided for participants as they were walking. Performance was quantified as the distance (meters) covered in 6 min. Although the 10-m adaptation of the 6MWT may result in shorter covered distances in healthy older adults due to the greater number of turns required [22] and limits the ability to compare to normative data that has used longer course lengths, it is considered a valid alternative [23] and has shown high test–retest reliability in clinical populations [24]. In this study the same course length was used for both timepoints, allowing comparison of performance across time.

The Timed Up and Go [19] test is a measure of overall physical function and falls risk which has demonstrated fair test–retest reliability in community-dwelling older adults (ICC = 0.74 for two testing sessions, 12 weeks apart in a previous study) [25]. During this task, participants were seated on a chair, with a marked line positioned three meters in front of them. Participants were timed to assess how long it took to arise from the chair, walk to the line, return to the chair, and sit down again. Performance was quantified as seconds taken to complete the course.

### Measures of psychosocial wellbeing

Psychosocial wellbeing was assessed at baseline and reassessment using the Friendship Scale, Mental Health Continuum (short form), and EQ-5D-5L.

The Friendship Scale [26] was used as a measure of social connectedness. The scale asks participants to rate the frequency of feelings over the past four weeks in response to five items, including: ‘I found it easy to get on with other people’ (item 1); ‘I felt lonely’ (item 2); ‘I had someone to share my feelings with’ (item 3); ‘I found it easy to make contact with people’ (item 4); ‘I felt I was a burden to people’ (item 5). For each item, participants provided a frequency rating (and subsequent score, in brackets) of ‘almost always’ (1), ‘most of the time’ (2), ‘about half the time’ (3), ‘occasionally’ (4), or ‘not at all (5). Items 2 and 5 were reverse scored. A total score was calculated by summing scores from all items (total score ranging from 5–25), where higher scores indicate greater feelings of social connectedness and lower scores being indicative of social isolation. The Friendship Scale has good reliability (Cronbach’s α = 0.83) and concurrent discriminant validity in older adult populations [27].

The Mental Health Continuum [28] is a 14-item questionnaire that measures aspects of mental wellbeing with high internal validity and moderate test–retest reliability in adults [29]. Each item required a score indicating frequency of feelings (and subsequent score, in brackets): ‘never’ (0), ‘once or twice’ (1), ‘about once a week’ (2), ‘about 2 or 3 times a week’ (3), ‘almost every day’ (4) and ‘every day’ (5). Three sub-scores were created to reflect *emotional* wellbeing (cluster 1: items 1–3, score out of 15), *social* wellbeing (cluster 2: items 4–8, score out of 25), and *psychological* wellbeing (cluster 3: items 9–14, score out of 30). Higher scores indicate better mental health. For this study, participants’ emotional, social, and psychological sub-scores were compared from baseline to reassessment.

The EQ-5D-5L is a measure of health-related quality of life [30] that has demonstrated excellent psychometric properties (i.e., reliability and validity) across a diverse range of populations (see recent review by Feng et al. [31]). The first five questions provided an indication as to whether individuals were experiencing problems with five dimensions of everyday life (mobility, self-care, usual activities, pain/discomfort, and anxiety/depression). Participants rated their level of difficulty with each item (and subsequent score, in brackets): ‘no problems’ (1), ‘slight problems’ (2), ‘moderate problems’ (3), ‘severe problems’ (4), or ‘extreme problems’ (5). Further, participants were asked to complete the EuroQol Visual Analog Scale (EQ-VAS), which required participants to provide a rating of today’s health from 0–100 (higher scores indicating better health).

### Qualitative interviews

Following the emergence of the COVID-19 pandemic, changes were made to the Daily Moves program to ensure it adhered to relevant national, state, and local health guidelines for participant safety. Daily Moves participants who had consented to participating in the research evaluation were contacted by staff and invited to participate in a semi-structured interview to discuss their experiences of Daily Moves so far, the impacts of the pandemic on their wellbeing, and their ongoing confidence and motivation to remain active.

Semi-structured interviews were held via phone or video conferencing (with audio recording) and recordings were transcribed verbatim. An interview guide was created to direct the interviews. Open-ended questions included asking participants to reflect on their experience of the Daily Moves program, their perceived activity levels and engagement in activities and wider supports before and after joining, and their motivation for continuing throughout the program despite COVID impacts.

Interviews were scheduled between December 2020 to February 2021 (inclusive). At the time of interviews, South Australia had experienced one recent period of statewide lockdown (November 2020) and was under public health restrictions supporting social distancing, mask wearing, density restrictions and other public health measures.

### Data analysis

Quantitative data were analyzed using R (R Core Team, 2020). For each measure, paired samples t-tests were used to investigate if changes (baseline to reassessment) in physical and psychosocial outcomes were statistically significant (alpha set at *p* < 0.05). For non-parametric data which violated the Shapiro–Wilk assumptions of normality, Wilcoxon sign-ranked tests were used to investigate associations.

Qualitative data were analyzed using reflexive thematic analysis [32]. Recordings were transcribed verbatim by Rev transcriptionists. Following return of the transcripts a process of data verification (comparing transcripts to original audio), cleaning (clarifying inaudible moments of audio) and familiarization was undertaken before coding and thematic analysis began. Data were manually coded using Microsoft Word and Excel to identify and refine themes. Coding and thematic analysis was primarily undertaken by MH with wider consultation with AC as early themes were identified and refined. Analyses used a realist perspective, whereby participants’ discussions were viewed as insights into their direct experiences within the program.

## Results

### Sample demographics

Of the 226 *Daily Moves* participants who completed baseline testing, 94 completed reassessment testing sessions, with a sub-sample of these participants providing consent for their data to be published. Thus, the current study included 69 older adults (mean age at baseline = 73.9 ± 5.6 years; 19 male) from six Adelaide council areas: Unley (*n* = 16); Walkerville (*n* = 6); Burnside (*n* = 17); Norwood Payneham St Peters (*n* = 7); Campbelltown (*n* = 19); and Prospect (*n* = 4). There was an average of 8.7 ± 1.0 months between baseline and reassessment visits.

### Physical activity engagement

**Fig. 2:**
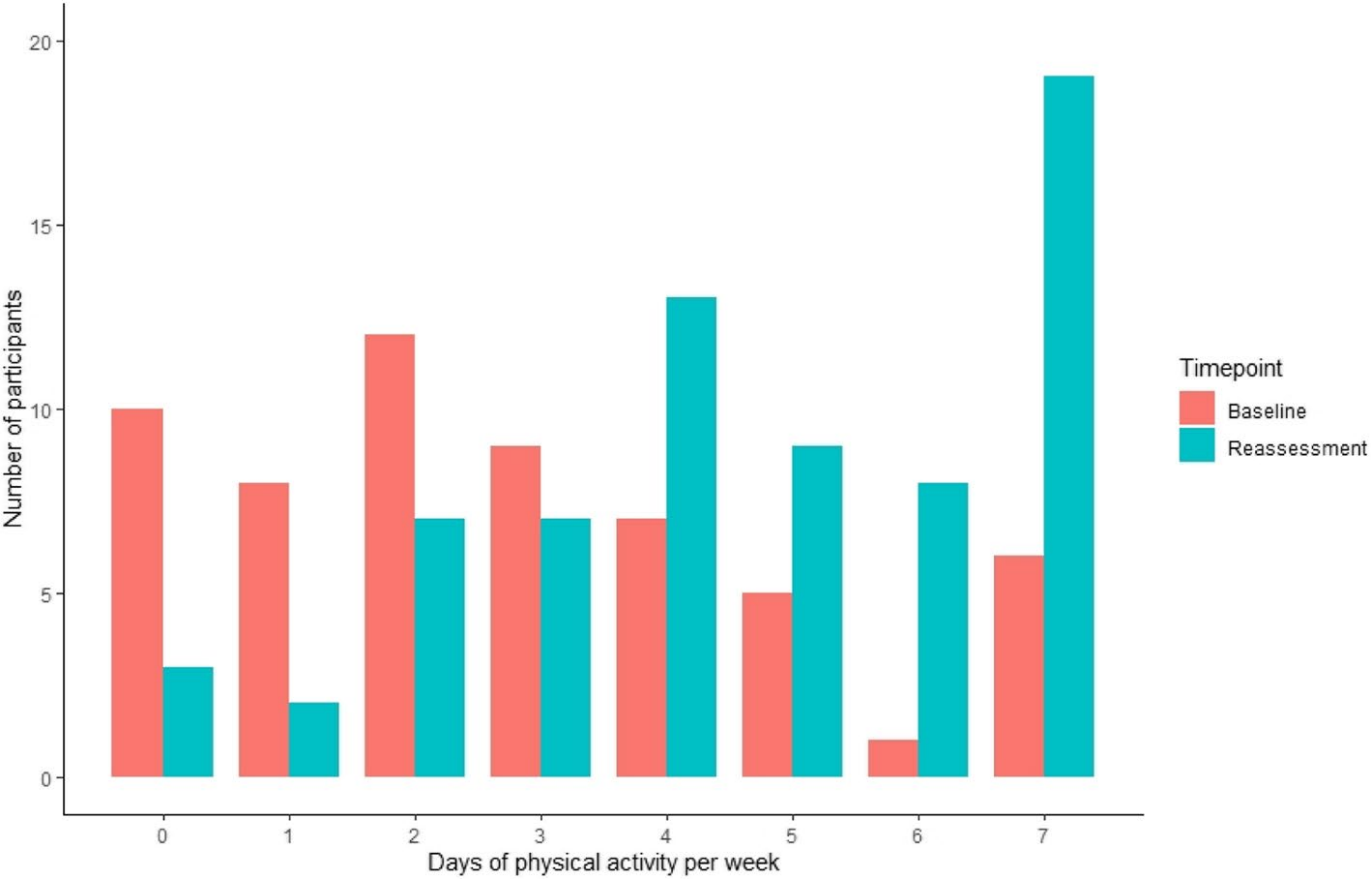
Physical activity engagement in Daily Moves participants

At baseline, participants reported engaging in 30 min of physical activity on 2.8 ± 2.1 of the past 7 days. This significantly increased to 4.6 ± 2.0 days at reassessment (t(56) = -4.3, *p* < 0.001, 95% CI = -2.4, -0.90; mean difference = 1.8 days) (Fig. 2)..

Figure 2 displays the number of Daily Moves participants who reported engaging in 0 to 7 days of physical activity in the past 7 days, at baseline and reassessment timepoints. Note: sample size at baseline was 58; sample size at reassessment was 68.

### Physical function and performance

Daily Moves participants improved across several physical function and performance measures from baseline to reassessment (Table 1). Improvements were statistically significant for right hand grip strength (t(66) = -2.21, *p* = 0.03, 95% CI = -2.4, -0.12), left hand grip strength (t(68) = -3.48, *p* < 0.001, 95% CI = -2.8, -0.76) and Timed Up and Go (t(68) = 8.1, *p* < 0.001, 95% CI = 1.0, 1.6). There were no statistically significant changes in performance on the Unipedal Stance test for either test condition (Wilcoxon sign-ranked test: eyes open best attempt, *p* = 0.43; eyes closed best attempt: *p* = 0.28), or the 6MWT (Wilcoxon sign-ranked test, *p* = 0.06).

**Table 1:** Physical wellbeing and performance scores

		**Baseline**		**Reassessment**			
	*n*	Mean	SD	Mean	SD	Mean diff	*p*
Grip Strength (best RH) (kg)	67	26.4	10.3	27.7	9.4	1.3	0.03*
Grip Strength (best LH) (kg)	69	24.2	9.5	26.0	9.8	1.8	< 0.001*
Unipedal Stance (best open) (sec)	65	29.3	17.1	31.0	17.3	1.7	0.43^a^
Unipedal Stance (best closed) (sec)	49	5.0	5.9	5.6	6.5	0.6	0.28^a^
Timed Up and Go (sec)	69	9.0	2.7	7.7	2.3	-1.3	< 0.001*
6-Minute Walk Test (sec)	67	408.5	91.5	436.3	116.1	-27.8	0.06^a^

*RH* right hand, *LH* left hand, *kg* kilograms, *sec* seconds, *mean diff* differences between mean values at baseline and reassessment

^a^denotes relationships that were tested using Wilcoxon sign-ranked tests (for non-parametric data which violated Shapiro–Wilk assumptions of normality)

^*^denotes statistical significance (*p* < .05)

### Psychosocial wellbeing

#### Friendship scale

All pairwise comparisons were tested for statistical significance using Wilcoxon sign-ranked tests. From baseline to reassessment, there were no significant changes in responses to item 1 (mean difference = -0.04, *p* = 0.83), item 2 (mean difference = 0.04, *p* = 0.87), item 3 (mean difference = 0.10, *p* = 0.55), item 4 (mean difference = 0.15, *p* = 0.06), or item 5 (mean difference = 0.08, *p* = 0.35) on the Friendship Scale. Total social isolation scores at baseline ranged from 5–16, with maximum scores increasing slightly at reassessment (range = 5–18). The changes in total social isolation scores from baseline to reassessment were not statistically significant (Wilcoxon sign-ranked test, *p* = 0.32).

#### Mental health continuum

There were no significant changes from baseline to reassessment for mental health continuum cluster 1 (Wilcoxon sign-ranked test, *p* = 0.86), mental health continuum cluster 2 (t(67) = -0.77, *p* = 0.44, 95% CI = -1.37, 0.60), or mental health continuum cluster 3 (Wilcoxon sign-ranked test, *p* = 0.61) (Table 2).

**Table 2:** Psychosocial wellbeing scores

		**Baseline**		**Reassessment**			
	*n*	Mean	SD	Mean	SD	Mean diff	*p*
Total social isolation score (FS)	65	8.2	2.8	7.8	2.8	-0.4	0.32^a^
MHC cluster 1	67	12.3	2.4	12.3	2.0	0.0	0.86^a^
MHC cluster 2	68	16.2	4.4	16.6	5.7	0.4	0.44
MHC cluster 3	67	23.0	5.2	23.6	4.9	0.6	0.61^a^
EQ-VAS (health today)	64	75.4	14.7	79.5	13.9	4.1	0.09^a^

*MHC* Mental Health Continuum, *EQ-VAS* EuroQol Visual Analog Scale, *FS* Friendship Scale, *mean diff* differences between mean values at baseline and reassessment

^a^denotes relationships that were tested using Wilcoxon sign-ranked tests (for non-parametric data which violated Shapiro–Wilk assumptions of normality)

#### EQ-5D-5L

From baseline to reassessment, there were no statistically significant changes in mobility (mean difference = 0.05, *p* = 0.53), self-care (mean difference = 0.04, *p* = 0.67), usual activities (mean difference = -0.10, *p* = 0.33), pain/discomfort (mean difference = -0.03, *p* = 0.87) or anxiety/depression (mean difference = 0.02, *p* = 0.62) (Additional File 1). Similarly, there were no significant changes in EQ-VAS ratings from baseline to reassessment (*p* = 0.09) (Table 2).

### Qualitative findings

Twenty-one participants agreed to be interviewed about their experiences of Daily Moves and how COVID-19 impacted their daily life. Participants were predominantly female (*n* = 17) and ranged in age from 66 to 91 years. Most participants had been engaged with the Daily Moves program for over 12 months at the time of interview (*n* = 16), with others having joined more recently because of the pandemic (*n* = 5). Following thematic analysis, three main themes were identified from the data. Participants reported the Daily Moves program to be *tailored, flexible and connected;* that the program *supported discipline and routine;* and that it *challenged to try new things*.

#### Tailored, flexible and connected

Participants highly valued the tailored approach that Daily Moves offered them and highlighted it as a point of difference compared to their experiences of other programs. Many participants who joined the program reported having at least one health concern (~ 53% of participants included in the evaluation), which was often a motivation to join. Across their engagement with Daily Moves, it was common for participant’s health conditions to fluctuate, resulting in necessary changes to a participant’s planned activity. Participants were empowered by the ability to adapt their program to suit their needs during these times.

“I still wanted to stay fit and I want to strengthen my upper body, and I find the gym situation isn’t suitable for me anymore” (Female, 77, Burnside, joined during COVID-19 pandemic).

“Well, I was doing all the exercises in the book that you gave me. And I'd decided I would get out and walk more. But I was finding it was getting really hard… Then I had to have the surgery and that put a stop to everything. So, I’ve finally got back to walking” (Female, 74, Campbelltown, joined during COVID-19 pandemic).

“There were a couple of videos that … Daily Moves people sent. And there was a couple that were not my level… Different programs there. I picked out things that suited me” (Female 71, Campbelltown, joined before COVID-19 pandemic).

Daily Moves participants reported feeling supported to choose an approach that suited their needs and situation. Participants were offered wider support throughout the program, including via telephone during the height of the pandemic, which was highly valued if not always something the participant felt they personally required.

“Well, I didn’t get all that many [phone calls] because I didn’t seek them. They were there to be had for sure” (Female, 92, Unley, joined before COVID-19 pandemic).

“Well, Daily Moves, they’ll keep me up to date mostly by phone” (Male, 76, Burnside, joined before COVID-19 pandemic).

“And I did get a call a couple of times from them. [Staff] actually did, I haven’t taken him up on it, but he did actually say if we wanted to, he would come to our house and show us a few things we could do in the house from an exercise point of view” (Male, 72, Campbelltown, joined before COVID-19 pandemic).

In addition, the ability to be active in a way that suited their individual needs and preferences while also remaining connected to the wider program and supports was important for many participants.

“I decided I like to walk by myself rather than in a group because you’re pushed a little bit more in a group, but that's not necessarily what I needed. I need to just take up my own pace, stop and talk to people with their dogs, and do it more as a sort of a ... It is a social thing” (Female, 77, Burnside, joined during COVID-19 pandemic).

Connections to community offered as part of the program were also highly valued. In some cases, the program provided opportunity for participants to reconnect to experiences they had once enjoyed but had to withdraw from. The program remaining connected to the local area was also important to participants and supported their ongoing engagement in activities.

“I’d moved over here and I didn't know anybody, and I thought I’d just get started on some things… I started with the heart group. I hadn’t intended to do that, but I just wanted to meet people” (Female, 71, Campbelltown, joined before COVID-19 pandemic).

#### Supported discipline and routine

Despite the COVID-19 pandemic, participants regularly reported feeling a sense of discipline and commitment to the program having established a regular routine for themselves around their wider community and social commitments. Participants were also encouraged to consider smaller opportunities across their day where they could engage in intermittent bouts of activity.

“That 20 minutes before I cook dinner, I’d make sure if I hadn’t done my few exercises, I will do it…. I think I do more intermittent things like when I’m boiling the kettle, I might do some leg raises or some pushes against the kitchen bench” (Female, 66, Walkerville, joined before COVID-19 pandemic).

Participants reported an unavoidable decrease in some types of physical activity due to COVID-19 restrictions. However, participants were able to adapt information and resources provided on home exercises to suit their needs during this time.

“No, because I was able to do the exercises at home and the pool would have stopped but the exercises at home … COVID didn’t hinder that at all.” (Female, 79, Unley, joined before COVID-19 pandemic).

“So no, but from the point of view of having to adapt to find different ways of exercise, that was a bit of a mental challenge to suddenly think, “Okay, I’ve got to keep exercising. How do I do it?” (Female, 72, Unley, joined before COVID-19 pandemic).

Participants commitment to the program remained high even after the pandemic restrictions were imposed. Commonly this was reported as being partially due to the discipline and accountability instilled by the program (related to regular assessment sessions) and supportive check-in phone calls that were offered.

“They encouraged me to come to be reassessed, when I really didn’t think I had [improved]...I thought I’d be shamed into finding I was so unfit compared to the last time, which didn’t look real good. But I think I measured up pretty much the same. It was really encouraging” (Female, 71, Campbelltown, joined before COVID-19 pandemic).

“It has for me, very much so. We do the exercises that we’ve been set three times a week… We’re being very diligent with our exercises set by [staff]. And I’ve been doing the dancing” (Female, 70, Norwood Payneham and St Peters, joined during COVID-19 pandemic).

“From the outset, knowing that someone was going to ring and checkup, was actually a very good discipline for me. Now I almost don’t need that discipline, but the first weeks that was really important, because that’s when you tend to, or I tend to fall by the wayside” (Female, 71, Prospect, joined before COVID-19 pandemic).

#### Challenged to try new things

Throughout the Daily Moves program participants were encouraged to try new and different physical activities and exercises that they may be interested in, or to view their body function in a new light. The physical activity suggestions and information provided in response to physical assessment results were highly motivating for participants and encouraged them to continue to challenge themselves to improve their activity further.

“To improve other aspects of, I suppose my physical condition, but I increased from doing laps of the local Oval [from] just walking, to now I jog sort of … I’ve increased the tempo a little bit” (Male, 72, Campbelltown, joined before COVID-19 pandemic).

“By having these weekly phone calls and then I was looking forward to my next assessment... And I had actually improved a bit. So that was good” (Female, 79, Norwood, Payneham and St Peters, joined before COVID-19 pandemic).

In addition, the wide range of resources provided through the regular newsletter and Daily Moves program partners offered opportunities to try new physical activities or learn new skills in a safe environment. Some participants were frustrated by the impact of COVID-19 on “come and try” sessions for new physical activities. However, participants reported being encouraged to find alternatives they could adjust within the boundaries of their own home or safely within their local neighbourhood.

“She used to send ideas that we could, well, I don’t think anyone could keep up with the number of options that she used to give. It was just so good” (Female, 92, Unley, joined before COVID-19 pandemic).

“I was determined and I wanted to learn how to use it [park-based gym equipment] and I want to go back to that space and do it, because it's such a beautiful space … for me it was a 10-minute walk beforehand to warm up, right by the riverbank and it's just beautiful” (Female, 71, Prospect, joined before COVID-19 pandemic).

[Staff] was emailing… There was a couple of things I said I was interested in doing. I actually found that really, really valuable… Konga class, which is like a dance class…. I started doing it with [instructor] at Unley. It challenges you and makes you use different muscles. You use different parts of your brain” (Female, 72, Unley, joined before COVID-19 pandemic).

## Discussion

This study presents a mixed-methods evaluation of Daily Moves, a community-based program which aimed to increase participation in physical activity among older adults living in eastern Adelaide, South Australia. Due to timing, the program ran almost entirely during the COVID-19 pandemic with varying levels of public health restrictions impacting activities across this time. Globally there has been a trend of decreased physical activity participation following the outbreak of the pandemic, due to public health measures, lockdowns, and restrictions on group activities [33]. Despite this, participants who remained enrolled in the Daily Moves program (i.e., attended a reassessment session) reported an increase in physical activity levels, measured as the number of days in the past week that they had participated in 30 min of activity. Further, there were improvements on several measures of physical function and no significant declines in psychosocial wellbeing. Qualitative findings gave further context to the changes in activity that occurred during the pandemic and provided insight into program features that supported participants to remain engaged with the program.

Participants reported an increase in the number of days that they had engaged in physical activity for 30 min or more (from 2 to 4 days per week) following participation in Daily Moves. This finding supports previous studies demonstrating the effectiveness of community-based physical activity programs for older adults [34, 35]. However, an important difference between previous programs and Daily Moves was the “light-touch”, self-directed nature of the program. That is, rather than providing a structured, one-size-fits-all intervention, the Daily Moves program increased participants’ awareness of activities and facilities available in their local area and allowed autonomy in selecting activities that suited their needs and preferences. Taken together, this finding provides preliminary evidence for the effectiveness of community-based interventions and supports the notion that encouraging older adults to find activities that they enjoy is beneficial.

Following the Daily Moves program, participants in this evaluation experienced improvements in Grip Strength (left and right) and Timed Up and Go test scores and maintained performance on the Unipedal Stance test and 6-Minute Walk tests (i.e., no significant decreases). In other words, participants improved their physical endurance and muscular strength, and did not experience a significant decline in balance over the course of the program. Muscular strength, mobility and endurance are associated with independence and quality of life in older adults, however aging is typically associated with a decrease in these indicators of physical health and function, as well as a decrease in physical activity which exacerbates physical decline [36]. As such, functional improvements that result from increasing physical activity levels are important and impactful outcomes for older adults following interventions, as they may have flow-on effects for quality of life. Our findings provide additional evidence that community-based programs like Daily Moves can facilitate important changes in physical function outcomes that have been linked to falls risk, morbidity, and mortality in older adults.

There were no significant changes in any psychosocial wellbeing assessments following the Daily Moves program. This somewhat contradicts findings of previous research that reported improvements in psychological and psychosocial wellbeing outcomes following participation in community-based physical activity programs [37]. Given the onset of the COVID-19 pandemic during the Daily Moves program, the maintenance of psychosocial wellbeing (i.e., no significant declines) may be interpreted as a positive outcome of the program. Moreover, our findings reflect a previous study which reported that older adults with higher physical activity levels were less depressed than their sedentary counterparts while COVID-19 social distancing restrictions were in place [38]. Further, evidence obtained during the first year of the COVID-19 pandemic indicated that older adults were less susceptible to declines in mental health compared to young adults [39]. Participants included in this evaluation scored well on psychosocial wellbeing measures at baseline, and thus may have been less susceptible to declines in mental health due to the pandemic [39].

Although no changes in quantitative psychosocial wellbeing measures were observed, participants reported a range of psychosocial benefits during the qualitative interviews. These included a sense of connectedness to the program and Daily Moves staff, building new or expanded friendships with others in shared group activities, and benefits of physical activity to facilitate their wider engagement in community-based settings. Participants who preferred being active alone also reported changes in their intentions, experience, and commitment to be active. Often this involved engaging with their community. Participants also reported enjoying a variety of activities because of the program, some new and novel to them, and others that they were able to reintegrate into their lives. These findings highlight that despite no significant quantitative changes, participants themselves identified a range of positive psychosocial impacts of being involved with the Daily Moves program.

Several aspects of the Daily Moves program likely contributed to its effectiveness for those who remained engaged. Following the announcement of the COVID-19 pandemic, Daily Moves staff maintained regular contact with participants via phone calls to provide support, which were highly valued by participants. During interviews, participants highlighted that the program promoted connectedness between staff and participants and identified that the assessment sessions (during which they undertook tests of physical function and psychosocial wellbeing) provided accountability to remain in the program and maintain activity levels. Daily Moves provided access to professional staff (through baseline testing sessions) who identified potential areas of weakness and subsequently provided tailored programs for each participant. Importantly, Daily Moves was embedded within the community and encouraged participants to utilize local facilities to increase their physical activity engagement. The program therefore did not rely heavily on access to specific equipment or facilities, instead providing a flexible and personalized approach, which afforded participants the ability to choose what activities to engage with, taking into consideration each participants’ needs, preferences and accessibility requirements. Previous research has identified that flexible and choice-based approaches to physical activity programs in older adults promote uptake and scalability [9]. So more older adults can benefit from the Daily Moves program model, it is recommended that multiple local government councils collaborate to hire dedicated staff to support and identify available opportunities in those areas and promote them to older adults looking to be more active. Staff should also be allowed the opportunity to engage with in-home supports and promotion of physical activity, enabling older adults to engage more readily in daily activities in their own home.

The mixed methods approach to this study was a strength. This design allowed for both quantitative assessment of participants physical activity, psychosocial wellbeing and physical function to be considered while also reviewing the items within the program design that participants found most valuable to engage with. There are several important limitations to consider. Firstly, Daily Moves was initially delivered as a community-based program, and recruitment into this research evaluation was secondary to participation in the wider program. Of the original 226 Daily Moves participants that participated in baseline assessments, only ~ 40% attended reassessment sessions, and of these, ~ 30% did not consent to having their data published. This is an important consideration as it is possible that the outcomes of those participants who attended the reassessment session and subsequently consented to the evaluation are not representative of the outcomes and experiences of the remaining Daily Moves participants. This may have inflated the benefits seen in physical function outcomes and physical activity levels reported in the current study. Further, given the explanatory sequential mixed-methods approach, having no control group limits our ability to determine cause-and-effect. In addition, multiple student assessors were used to collect objective data, with no inter-rater reliability tests conducted. The COVID-19 pandemic also impacted the planned assessment timeframes for participants, while assessments were conducted as close as practical (considering public health restrictions) to the scheduled time frames. Finally, the length of the 6MWT was reduced due to space constraints, but although the 10-m distance may result in shorter distances covered (compared to the standard 30-m course) [22], it is considered a valid alternative [23]. Results should be interpreted with these limitations in mind.

## Conclusions

Daily Moves had a positive impact on participants who continued their engagement with the program over approximately 9 months, particularly for physical activity levels and physical function outcomes. The program was viewed positively by participants, with the support provided by staff amidst the COVID-19 pandemic acknowledged as a main driver for its success during qualitative interviews. This evaluation demonstrates that physical activity programs embedded within the community can provide flexible and tailored recommendations to participants, and that this approach can promote positive change in important indicators of health in older adults.

## Supplementary Information

**Additional file 1.** 

## Data Availability

The datasets used and/or analyzed during the current study are available from the corresponding author on reasonable request.
