# Supplementary material for: Mixed-methods evaluation of Daily Moves, a community-based physical activity program for older adults
Source: BMC Geriatr. 2022 Nov 12;22:853. doi: 10.1186/s12877-022-03567-6 (PMC9655805; doi:10.1186/s12877-022-03567-6)
Supplement: Supplementary file 1 — Additional file 1. [file 12877_2022_3567_MOESM1_ESM.docx]

| **EQ-5D-5L Dimension** | **Rating** | **Baseline, n (%)** | **Reassessment, n (%)** | **p-value** |
| --- | --- | --- | --- | --- |
| Mobility | No problems | 40 (60.6) | 42 (63.6) | 0.53^a^ |
|  | Slight problems | 11 (16.6) | 14 (21.2) |  |
|  | Moderate problems | 13 (9.7) | 9 (13.6) |  |
|  | Severe problems | 1 (1.5) | 1 (1.5) |  |
|  | Unable to walk about | 1 (1.5) | 0 (0.0) |  |
| Self-care | No problems | 62 (93.9) | 63 (95.4) | 0.67^a^ |
|  | Slight problems | 2 (3.0) | 3 (4.5) |  |
|  | Moderate problems | 2 (3.0) | 0 (0.0) |  |
|  | Severe problems | 0 (0.0) | 0 (0.0) |  |
|  | Unable to wash or dress | 0 (0.0) | 0 (0.0) |  |
| Usual activities | No problems | 43 (65.1) | 37 (56.0) | 0.33^a^ |
|  | Slight problems | 15 (22.7) | 22 (33.3) |  |
|  | Moderate problems | 7 (10.6) | 8 (12.1) |  |
|  | Severe problems | 1 (1.5) | 1 (1.5) |  |
|  | Unable to do usual activities | 0 (0.0) | 0 (0.0) |  |
| Pain/discomfort | No pain/discomfort | 15 (23.8) | 15 (23.8) | 0.87^a^ |
|  | Slight pain/discomfort | 35 (55.5) | 34 (53.9) |  |
|  | Moderate pain/discomfort | 11 (17.4) | 11 (17.4) |  |
|  | Severe pain/discomfort | 2 (3.1) | 3 (4.7) |  |
|  | Extreme pain/discomfort | 0 (0.0) | 0 (0.0) |  |
| Anxiety/depression | Not anxious/depressed | 34 (56.6) | 38 (63.3) | 0.62^a^ |
|  | Slightly anxious/depressed | 20 (33.3) | 14 (23.3) |  |
|  | Moderately anxious/depressed | 5 (8.3) | 7 (11.6) |  |
|  | Severely anxious/depressed | 1 (1.6) | 0 (0.0) |  |
|  | Extremely anxious/depressed | 0 (0.0) | 1 (1.6) |  |

**Additional File 1**

EQ-5D-5L scores

^a^denotes relationships that were tested using Wilcoxon sign-ranked tests (for non-parametric data which violated Shapiro-Wilk assumptions of normality).
